# Creating the Right Sort of Ship to Achieve Integrated Care: A Response to Recent Commentaries

**DOI:** 10.15171/ijhpm.2019.04

**Published:** 2019-02-02

**Authors:** Gill Harvey, Joanne Dollard, Amy Marshall, Manasi Murthy Mittinty

**Affiliations:** ^1^Adelaide Nursing School, University of Adelaide, Adelaide, SA, Australia.; ^2^Adelaide Medical School, University of Adelaide, Adelaide, SA, Australia.; ^3^Centre for Population Oral Health, Adelaide Dental School, Adelaide, SA, Australia.

We welcome the opportunity to respond to the interesting and insightful commentaries provided by Rod Sheaff^1^ and Axel Kaehne^2^ on our editorial *Achieving Integrated Care for Older People: Shuffling the Deckchairs or Making the System Watertight For the Future*?^3^ In this editorial, we reflected on whether the multiple attempts to improve integrated care for older people living with multi-morbidity were getting to the root of the problem, or merely *shuffling the deckchairs while the ship is sinking* to use the Titanic analogy. Our argument was that initiatives that focused on fixing parts of the system were more likely to be shuffling the deckchairs. In order to really stop the ship from sinking, our proposal was that we needed to focus on the following key issues: making sure that attempts at integrating care are firmly centred on older people themselves and their individual aims and expectations; paying attention to the processes of implementation and taking account of the complexity involved; and adopting co-production approaches that enable collaborative working amongst all the relevant stakeholders, with patients and their carers at the centre.

In their commentaries, Sheaff and Kaehne have offered two different perspectives on the issues raised. Sheaff adds to the Titanic metaphor, asking *What Kind of Ship?* and questioning whether the ship is broad enough in the way that services are currently configured. Drawing on a realist systematic review of recent research,^4^ Sheaff highlights some of the key mechanisms identified that support integrated care initiatives, for example, the presence of multi-disciplinary teams, care planning, IT support and organisational culture change. One suggestion to broaden the ship and enable the aforementioned mechanisms is to bring the diverse services that older people with multiple chronic conditions need into a single organisation. Sheaff argues this would help to remove many of the inter-organisational boundaries that impede care integration, including both inter-professional and inter-service boundaries.

Kaehne presents an alternative *cart and horse* metaphor, suggesting that too often attempts to improve integrated care put the cart before the horse. From Kaehne’s perspective, integrated care has remained mostly aspirational to date, not least because of a failure to view service integration from the patient’s perspective. Reasons put forward for perceived failed ‘solutions’ include: governance of a complex (health) system by a rationalistic approach to decision-making; a need to differentiate between organisational and social features of integration; defining integration in terms of what organisations do, rather than by the care that patients receive. Kaehne points out the misguided assumption that approaching integration from an organisational/service perspective will coincide with improved patient experience. In order to put the horse before the cart, Kaehne’s argument is that we need to start with the patient experience and work upwards to the organisational level.

Sheaff and Kaehne’s proposals are, to some extent, a juxtaposition: one advocating for a potential structural solution to remove the boundaries that impede integrated care; the other suggesting that the organisational approach risks putting the cart before the horse by not starting from the patient perspective. Reflecting on these commentaries, we offer the following observations. Firstly, in relation to Sheaff’s identification of the boundaries that impede care integration, these are clearly important and we would support the need to build the key mechanisms identified from the realist review. However, as Sheaff points out, integration has largely been studied at the horizontal level – for example, within a primary care setting – rather than vertical integration, such as that between acute and primary care services. Such vertical integration likely poses a different set of challenges, not least in a country such as Australia, where primary care is governed and funded at the national level and acute care at the state level. Equally, it is important to note that not all boundaries are structural, thus whilst creating a single organisation might remove some of the barriers to integration, it may not overcome more deep-seated cultural barriers, related to values and beliefs. The findings of the systematic review partly confirm this, suggesting that more favourable contexts for integrated care exist when there are past experiences of collaboration and workplace cultures of mutual respect amongst different professional groups.^4^

From the patient perspective, Kaehne argues that organisational and professional boundaries matter little to patients; their concern is for a seamless and smooth journey through the system. Our own and others’ research supports this viewpoint,^5,6^ also highlighting that patients want to be treated holistically, taking into account not just their health needs, but also functional and social issues. Like Kaehne, this led us to call for a patient-centred view of integrated care, where solutions are developed in a locally, contextually relevant and bottom-up way. This requires a degree of creativity, flexibility and responsiveness, which will undoubtedly be challenging, but is to be expected when tackling the so-called complex, *wicked* problems in society.^7^

In their commentaries, Sheaff and Kaehne have contributed excellent insights into two of the issues we raised in our editorial – that is, prioritising the voices and experiences of older people, and enabling collaborative working. However, we believe there is still the important issue of focusing on processes of design and implementation. This brings us back to our recommendation of co-production, to achieve the previous two aims of a patient-centred, collaborative approach, in a way that recognises the complexities and competing tensions involved. Returning to our analogy of the Titanic, while it is important that the ship is big enough and broad enough, it is even more crucial that we get the design right and sufficiently engage with the patients and carers who we intend to sail in it.

## Acknowledgements

We gratefully acknowledge funding from The Hospital Research Foundation that is supporting our research into improving the integration of care for older people.

## Ethical issues

Not applicable.

## Competing interests

Authors declare that they have no competing interests.

## Authors’ contributions

All authors have been involved in research that contributes to our views in this editorial. GH drafted the original version of the manuscript. All authors contributed revisions and approved the final version.

## Authors’ affiliations

^1^Adelaide Nursing School, University of Adelaide, Adelaide, SA, Australia. ^2^Adelaide Medical School, University of Adelaide, Adelaide, SA, Australia. ^3^Centre for Population Oral Health, Adelaide Dental School, Adelaide, SA, Australia.
